# Anti–interferon-γ autoantibody–associated adult-onset immunodeficiency with occult immunological abnormalities and sequential intracellular infections: a case report

**DOI:** 10.3389/fimmu.2026.1840699

**Published:** 2026-05-13

**Authors:** Xiran Zhou, Panjie Hu, Qingxia Fu, Huanchang Chen, Juan Pan, Endian Sun, Shiyi Shi, Jianzhong Ye, Deyi Zhao, Tieli Zhou, Lijiang Chen

**Affiliations:** 1 Wenzhou Medical University, Wenzhou, Zhejiang, China; 2 Department of Clinical Laboratory, The First Affiliated Hospital of Wenzhou Medical University, Wenzhou, Zhejiang, China; 3 School of Laboratory Medicine and Life Science, Wenzhou Medical University, Wenzhou, Zhejiang, China

**Keywords:** anti-interferon-γ autoantibodies, case report, intracellular infection, *Legionella pneumophila*, multiple infections, *salmonella enteritidis*, *Talaromyces marneffei*

## Abstract

Adult-onset immunodeficiency associated with anti-interferon-γ (IFN-γ) autoantibodies (AIGA) is a rare immune disorder that neutralizes IFN-γ activity and compromises defense against intracellular pathogens. It is predominantly reported in Southeast Asia and predisposes affected individuals to recurrent and multiple intracellular bacterial infections, which can be life-threatening. As a “silent disruptor,“ AIGA often go unnoticed due to their insidious nature, leading to a lack of sufficient clinical awareness. This report presents the case of a 59-year-old male patient from Wenzhou, Zhejiang Province, China, who experienced multiple severe infections over one year. These included disseminated bloodstream infection caused by *Salmonella enteritidis*, pneumonia caused by *Legionella pneumophila*, and osteomyelitis (an infection of bone and bone marrow caused by microbial pathogens) of the right scapula caused by *Talaromyces marneffei*, which are rarely observed consecutively in immunocompetent individuals. The patient exhibited no distinct immune phenotypic abnormalities, leading to the insidious nature of the disease. After receiving short-term targeted antimicrobial therapy, long-term rituximab treatment and prophylactic anti-infective therapy, the patient eventually recovered and was discharged, with regular follow-up to monitor AIGA titers. This case suggests that AIGA–associated immunodeficiency should be considered in adults presenting with unexplained or recurrent intracellular infections, even when their immunophenotypic features appear unremarkable. Prompt detection of AIGA is essential for establishing an accurate diagnosis and initiating appropriate treatment. Immunomodulatory therapy with rituximab, in combination with targeted antimicrobial therapy and close long-term follow-up, can achieve favorable clinical outcomes. Ongoing clinical experience and research are needed to establish standardized therapeutic strategies and determine the optimal treatment duration for this rare immunodeficiency.

## Graphical Abstract

**Figure:**
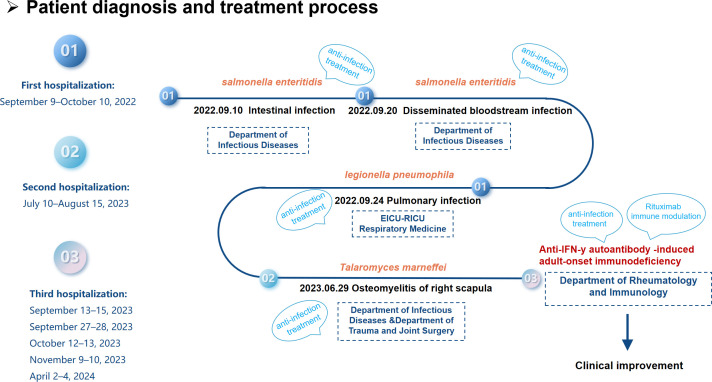

## 1 Introduction

Anti–IFN-γ autoantibody (AIGA)–associated adult-onset immunodeficiency (AOID) is an acquired autoimmune condition that phenotypically resembles immunodeficiencies caused by congenital defects in the IFN-γ signaling pathway (IL-12/IFN-γ axis), such as Mendelian susceptibility to mycobacterial disease. The immunodeficiency results from AIGA-mediated neutralization of IFN-γ, thereby impairing host immune responses and facilitating pathogen persistence (1).

As a potent immune activator, IFN-γ is the only type II interferon, primarily secreted by Th1 helper cells, CD8^+^ T cells, and natural killer (NK) cells in the immune system (2). AIGA will arise in genetically predisposed individuals in the setting of persistent antigenic stimulation and impaired immune tolerance. HLA class II–mediated antigen presentation, together with chronic infection, supports the activation of autoreactive T and B cells, permitting IFN-γ–reactive B cells to escape tolerance and undergo affinity maturation. This process results in the production of high-affinity, neutralizing IgG antibodies targeting IFN-γ (3). Excessive AIGA production leads to effective neutralization of IFN-γ (4). This disrupts immune signaling pathways, such as STAT1 phosphorylation, and inhibits the effective binding of IFN-γ to its receptor, ultimately impairing immune cell function. As a result, affected individuals exhibit increased susceptibility to infections, developing immunodeficiency symptoms similar to those observed in advanced human immunodeficiency virus (HIV) infection (5). The affected patients are highly vulnerable to opportunistic infections, which can be life-threatening (6, 7).

Currently, clinical awareness of AIGA remains insufficient, posing significant challenges for early diagnosis. As a “silent disruptor, “ AIGA-induced clinical symptoms are atypical and difficult to categorize, making early recognition challenging. Additionally, studies suggest that high AIGA titers are strongly associated with poor infection outcomes (8), underscoring the importance of AIGA monitoring. Here, we report a case of recurrent and multiple infections caused by AIGA. The patient experienced a series of severe infections within one year, including disseminated bloodstream infection caused by *Salmonella enteritidis* (*S. enteritidis*), pneumonia caused by *Legionella pneumophila* (*L. pneumophila*), and osteomyelitis of the right scapula caused by *Talaromyces marneffei* (*T*. *marneffei*). The initial diagnosis did not raise clinical suspicion, and only after multiple rounds of anti-infective treatment was an underlying immunodeficiency considered. The patient was ultimately diagnosed with AOID by our hospital and subsequently received multiple rounds of rituximab therapy to suppress AIGA. This treatment resulted in effective disease control and a favorable prognosis. Given the high prevalence of AOID in certain regions, serum AIGA testing should be considered for patients presenting with recurrent or multiple infections within a short period, facilitating timely diagnosis and improved clinical outcomes. This report also explores treatment strategies and highlights the necessity of laboratory-based serum AIGA monitoring.

## 2 Case presentation

### 2.1 First hospital admission: progression from acute gastroenteritis to bacteremia and hospital-acquired pneumonia (September 9–October 10, 2022)

A 59-year-old male presented with a 4-day history of abdominal pain and watery diarrhea on September 9, 2022. He had a past medical history of hypertension (6 years) and diabetes mellitus (8 years). The patient had no history of immunosuppressive therapy, including corticosteroids or biologic agents. HIV testing was negative. There was no history of malignancy, hematological disorders, or recurrent/opportunistic infections prior to this episode. In addition, no significant occupational or environmental exposures were identified. No developmental abnormalities, intellectual disability, or dysmorphic features were observed in this patient.

**Figure 1:**
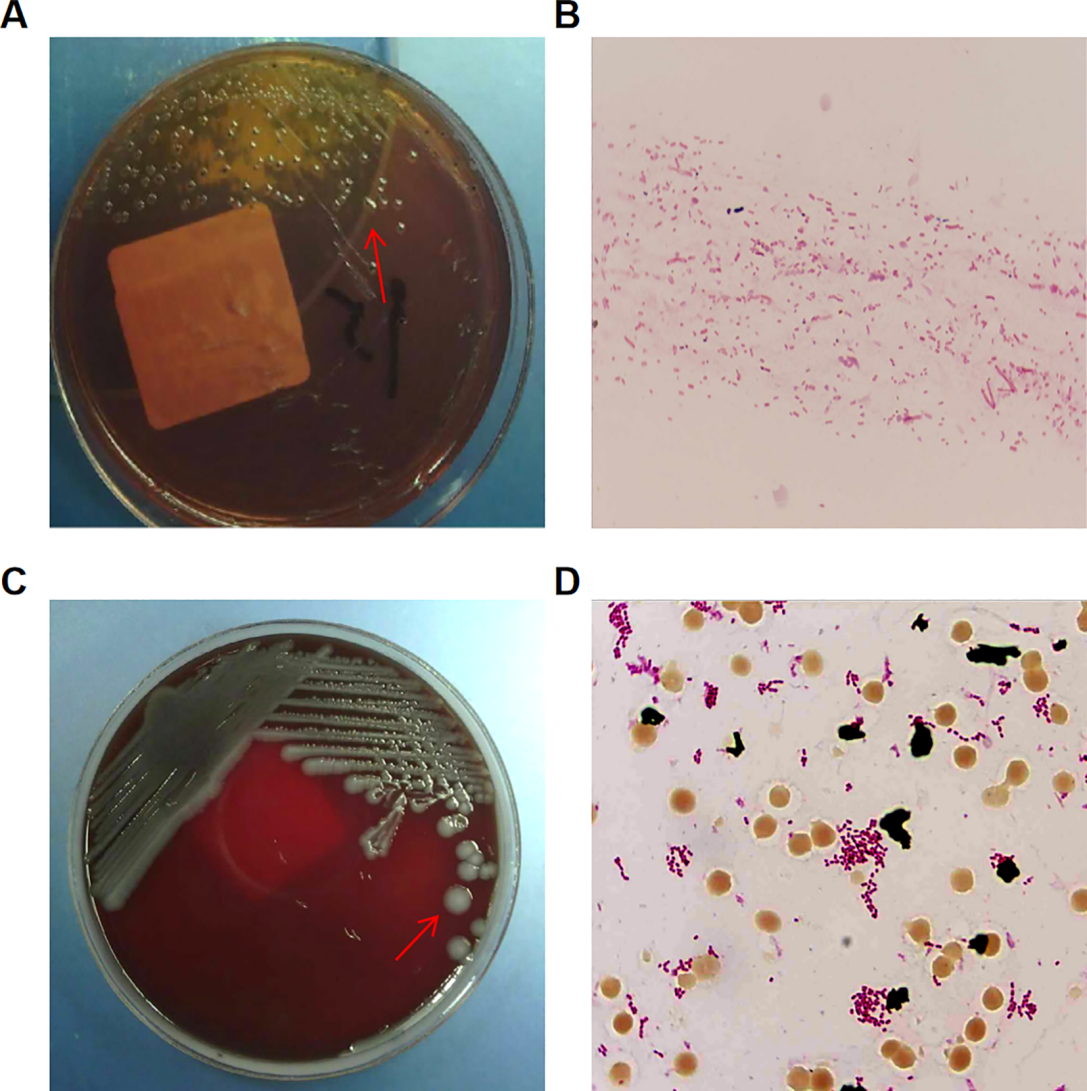
Pathogen identification of *Salmonella enteritidis* infection in this patient. **(A)** Fecal culture, colonies grown on *Salmonella Shigella* agar plates, the red arrow indicates the cultured *Salmonella enteritidis*; **(B)** Gram staining of the cultured colonies and microscopic observation (1000×); **(C)** Blood culture, colonies grown on Columbia blood agar plates, the red arrow indicates the cultured *Salmonella enteritidis*; **(D)** Gram staining of the cultured colonies and microscopic observation (1000×).

He reported recent consumption of potentially contaminated food and presented with yellow watery stools without melena or hematochezia, accompanied by vomiting. Prior to admission, laboratory tests at a local hospital showed markedly elevated inflammatory markers (hs-CRP 301 mg/L; neutrophils 77.1%), and empirical ceftriaxone partially relieved his symptoms. Infectious diarrhea was therefore considered the most likely diagnosis. At outpatient evaluation, acute gastroenteritis was suspected, and cefdinir was prescribed based on clinical presentation and laboratory findings (**Supplementary Tables S1**, **S2**); hospital admission was subsequently recommended for further management. Following admission, stool culture (It should be noted that stool bacterial culture were performed after the initiation of therapy, which may have affected the diagnostic yield) revealed colorless translucent colonies with black centers obtained on *Salmonella Shigella* agar plates, Gram staining microscopic observation revealed Gram-negative non-spore-forming bacilli (**Figures 1A, B**), and matrix-assisted laser desorption/ionization time-of-flight mass spectrometry (MALDI-TOF MS) identified them as *S. enteritidis*. The patient received intravenous cefoperazone-sulbactam combined with intestinal microbiota regulators, resulting in rapid improvement of diarrhea. Inflammatory markers progressively declined, with C-reactive protein (CRP) decreasing from 207.1 mg/L to 15.9 mg/L and Procalcitonin (PCT) from 1.72 ng/mL to 0.12 ng/mL. (**Supplementary Table S1**).

However, the patient developed a fever and a productive cough on September 18, 2022. Laboratory tests indicated elevated inflammatory markers, with a WBC of 15.15 × 10^9^/L, a neutrophil count of 12.88 × 10^9^/L, and a CRP level of 159.3 mg/L. On September 22, peripheral blood cultures were reported positive (14.9 hours), yielding medium-sized, round, smooth, moist, gray-white colonies (**Figure 1C**). Gram staining revealed gram-negative, non-spore-forming bacilli (**Figure 1D**). Serological identification showed A–F (+), O9+, Hg+, and Hm+, which is consistent with *Salmonella enterica* serovar Enteritidis according to standard Kauffmann–White classification. It consistent with disseminated *Salmonella enteritis* bloodstream infection.

**Figure 2:**
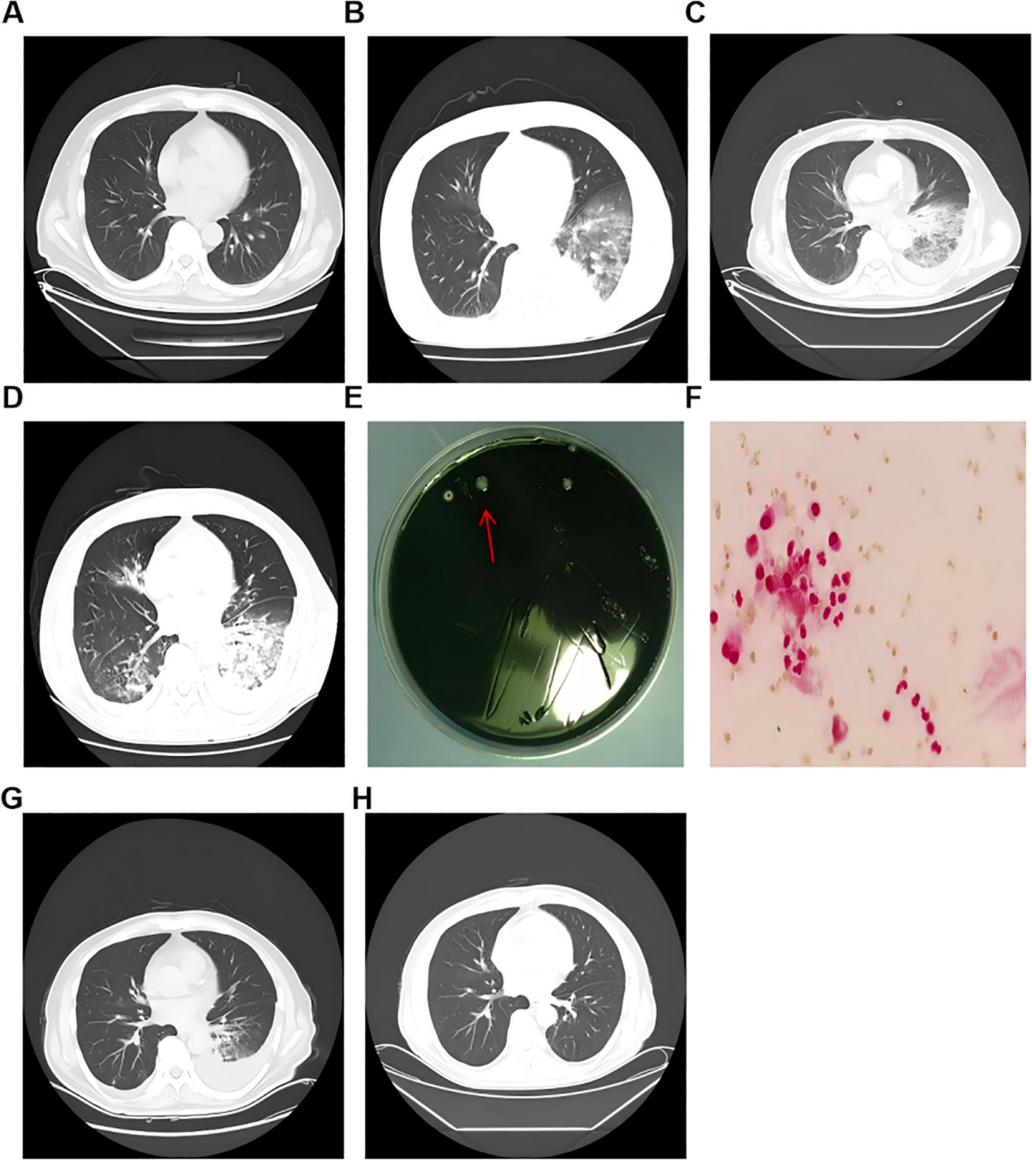
The results of alveolar lavage fluid testing and chest CT changes for the patient with *Legionella pneumophila* infection. **(A–D)** Chest CT images showing the progression of pulmonary infection, taken on September 9, September 20, September 22, and October 6, 2022, respectively. **(E)** Alveolar lavage fluid culture showing colony growth on *Legionella*-selective agar. The red arrow indicates the cultured *Legionella pneumophila*. **(F)** Gram staining of the cultured colonies observed under a microscope (1000×). **(G–H)** Chest CT images showing the improvement of pulmonary infection, taken on November 1 and November 24, 2022, respectively.

Meanwhile, a chest CT scan on the same day revealed a large area of consolidation and ground-glass opacity in the lower lobe of the left lung compared to September 9, indicative of secondary pulmonary infection (**Figures 2A, B**). The patient received imipenem for anti-infection treatment, ambroxol and acetylcysteine for expectoration, and compound methoxamine capsules for cough relief. However, the symptoms did not significantly improve, presenting with with dyspnea, orthopnea, tachypnea (40 breaths/min), and oxygen saturation fluctuating around 93%. The patient produced yellow-green sputum, and follow-up computed tomography (CT) revealed progression of pulmonary consolidation (**Figures 2C, D**). On September 24, a bedside bronchoscopy was performed, and secretions were collected for analysis. Buffered Charcoal Yeast Extract (BCYE) agar culture identified *L. pneumophila* (**Figure 2E**). Gram staining of the bronchoalveolar lavage fluid (BALF) showed no detectable pathogens (**Figure 2F**). DNA analysis (fluorescence-based PCR methods) confirmed the presence of *L. pneumophila* genes (**Supplementary Table S2**), and metagenomic next-generation sequencing (mNGS) further identified *L. pneumophila* (**Supplementary Table S3**). A definitive diagnosis of *L. pneumophila* was established.

The patient reported that sequential severe infections caused by two different pathogens imposed a significant physical and psychological burden. He was diagnosed with intestinal infection, bacteremia, and hospital-acquired pneumonia. Following combination therapy with imipenem and moxifloxacin, the body temperature normalized, microbiological cultures of stool, blood, and sputum became negative. Follow-up chest CT demonstrated significant resolution of pulmonary infiltrates (**Figures 2G, H**). The favorable clinical outcome did not prompt clinicians to consider the possibility of hidden diseases.

### 2.2 Second hospital admission: shoulder osteomyelitis caused by *Talaromyces marneffei* (June 27–August 15, 2023)

**Figure 3:**
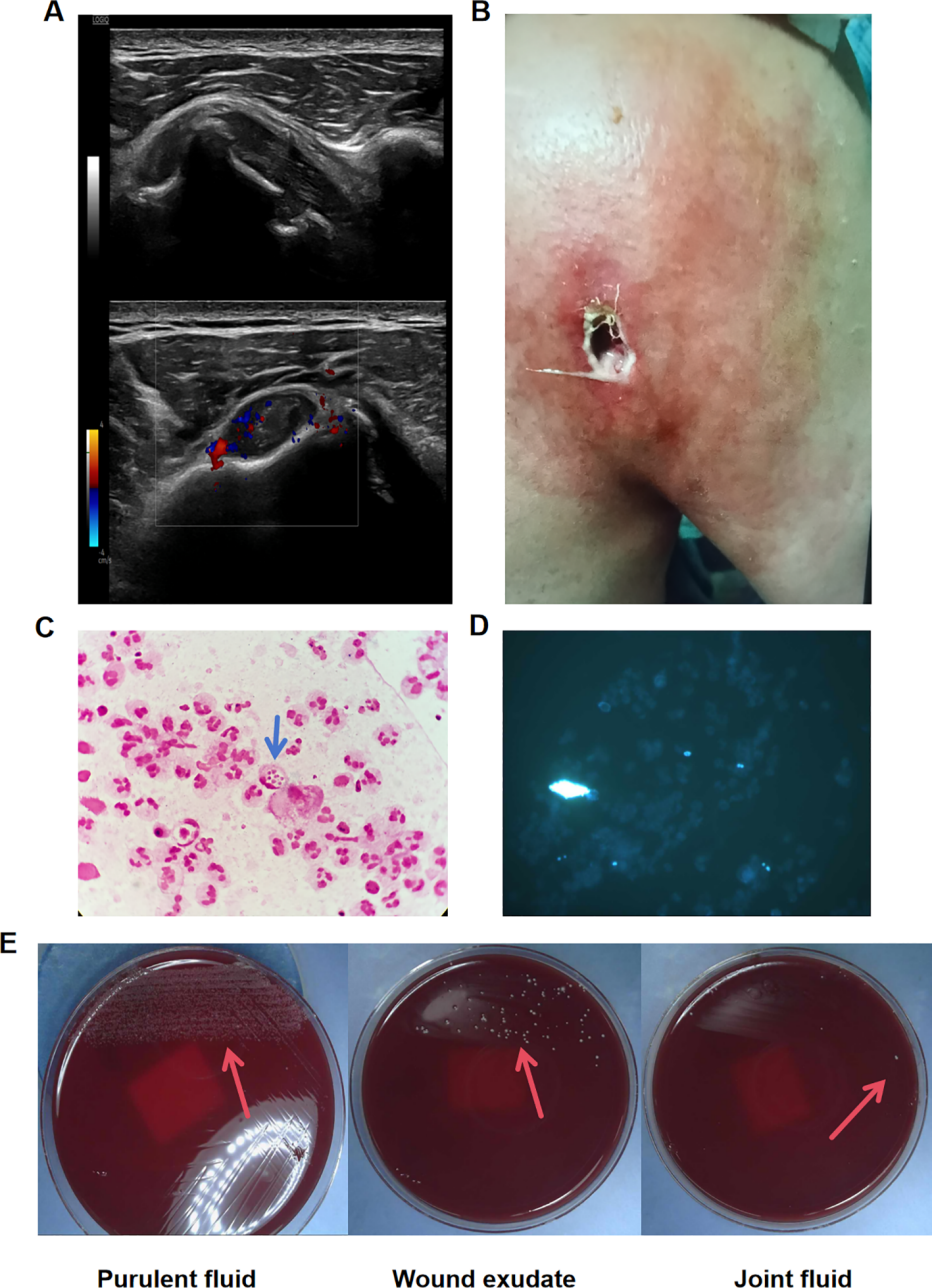
Examination results and pathogen identification of *Talaromyces marneffei* infection in the patient. **(A)** Ultrasound of the right shoulder. **(B)** Extensive infectious lesions with abscess formation in the soft tissue of the right shoulder joint. **(C)** Gram staining of the pus sample (1000×). **(D)** Fluorescence staining of the pus sample showing endospores with blue-violet fluorescence (500×). **(E)** *Talaromyces marneffei* isolated from pus, synovial fluid, and wound exudate after 48 hours of incubation at 37 °C. The blue arrow indicates the spores, the red arrow indicates the cultured *Talaromyces marneffei*.

On June 27, 2023, the patient was readmitted with complaints of restricted right shoulder movement for over 8 months and swelling and redness for more than 20 days. The patient reported recent sleep disturbance and used hypnotic medications intermittently. He denied a history of cardiovascular disease, chronic infectious diseases (including hepatitis or tuberculosis), allergies, prior surgeries, or blood transfusions. Previous antimicrobial and supportive treatments during prior hospitalization led to partial clinical improvement; however, the symptoms had developed insidiously following discharge from the respiratory department, with no identifiable precipitating event, and were characterized by progressive limitation of motion and weakness on abduction. Ultrasound of the right shoulder demonstrates extensive inflammatory changes, with irregular bony cortex and osteophyte formation. Abnormal soft tissue echogenicity with increased vascularity in the posterior shoulder suggests inflammatory involvement (**Figure 3A**). The symptoms persisted without improvement. At the time of admission, a 10×10 cm firm mass appeared on the posterior right shoulder, with skin desquamation, redness, swelling, heat, and tightness, but no discharge (**Figure 3B**), and relevant laboratory parameters are shown in **Supplementary Table S4**. Initial therapy with imipenem and linezolid was initiated for infection control.

During hospitalization, a shoulder abscess developed. Gram staining of the pus specimen shows numerous neutrophils, with some containing blunt-ended sausage-shaped fungal spores (indicated by blue arrows), and some spores have septa, representing a characteristic morphological feature of *T. marneffei* (**Figure 3C**). Fluorescence staining further highlighted abundant fungal elements, supporting the presence of fungal infection (**Figure 3D**). Cultures of both the joint fluid and wound exudate also grew *T. marneffei* (**Figure 3E**), and identification was confirmed by MALDI-TOF MS. Taken together, the characteristic microscopic morphology and concordant culture results from two clinically involved sites support *T. marneffei*. Based on these findings, antimicrobial therapy was adjusted to amphotericin B plus ceftriaxone on July 7. The patient developed a right shoulder abscess, and multidisciplinary consultation was obtained, which confirmed the indication for surgical intervention.

On July 12, arthroscopic debridement of the right shoulder abscess was performed to achieve infection control, followed by continued antimicrobial and anti-inflammatory therapy, including amphotericin B, ceftriaxone, and dexamethasone. However, On July 14, the patient’s blood creatinine increased to 292 μmol/L (reference range 44-97 μmol/L), indicating impaired renal function. Dexamethasone injection and amphotericin B injection were stopped, and voriconazole was initiated for infection treatment. The wound gradually improved thereafter (**Supplementary Figure S1**). The patient was discharged on August 15. The sequential occurrence of multiple serious infections alerted the doctors to the possibility of an underlying immune disorder.

The patient reported good adherence to treatment and satisfactory clinical outcomes. However, since August 29, 2023, he has intermittently experienced anxiety and sleep disturbances, which he attributed to recurrent infections.

### 2.3 Third hospitalization (five short readmissions): diagnosis and management of AOID (September 2023–April 2024)

The patient underwent five short readmissions between September 2023 and April 2024 for further immunological evaluation and treatment.

The patient, who was previously treated at our hospital in 2022 for *Salmonella* enteritis and *Legionella* pneumonia, was readmitted for right shoulder joint infection caused by *T. marneffei*. Over a short period, the patient developed multiple infections due to opportunistic pathogens, which is uncommon in immunocompetent individuals. This raised suspicion for an underlying immune dysfunction, prompting further screening for immune related diseases. Laboratory tests revealed negative results for a variety of autoimmune markers, including PCNA, anti-Ro-52 antibody, AMA-M2, ribonucleoprotein antibody, PM-SCL, ANA series, centromere antibodies, nucleosome antibodies, and anti-gad antibody, effectively ruling out systemic lupus erythematosus, Sjögren’s syndrome, polymyositis/dermatomyositis, and primary biliary cholangitis. Screening for autoimmune vasculitis-related antibodies (e.g., PANCA, MPO-ANCA, GBM, CANCA, PR3-ANCA) also returned negative. At the same time, no common causes of secondary immunosuppression, such as HIV infection or anticancer therapy, were identified, suggesting a possible unrecognized immunodeficiency.

**Figure 4:**
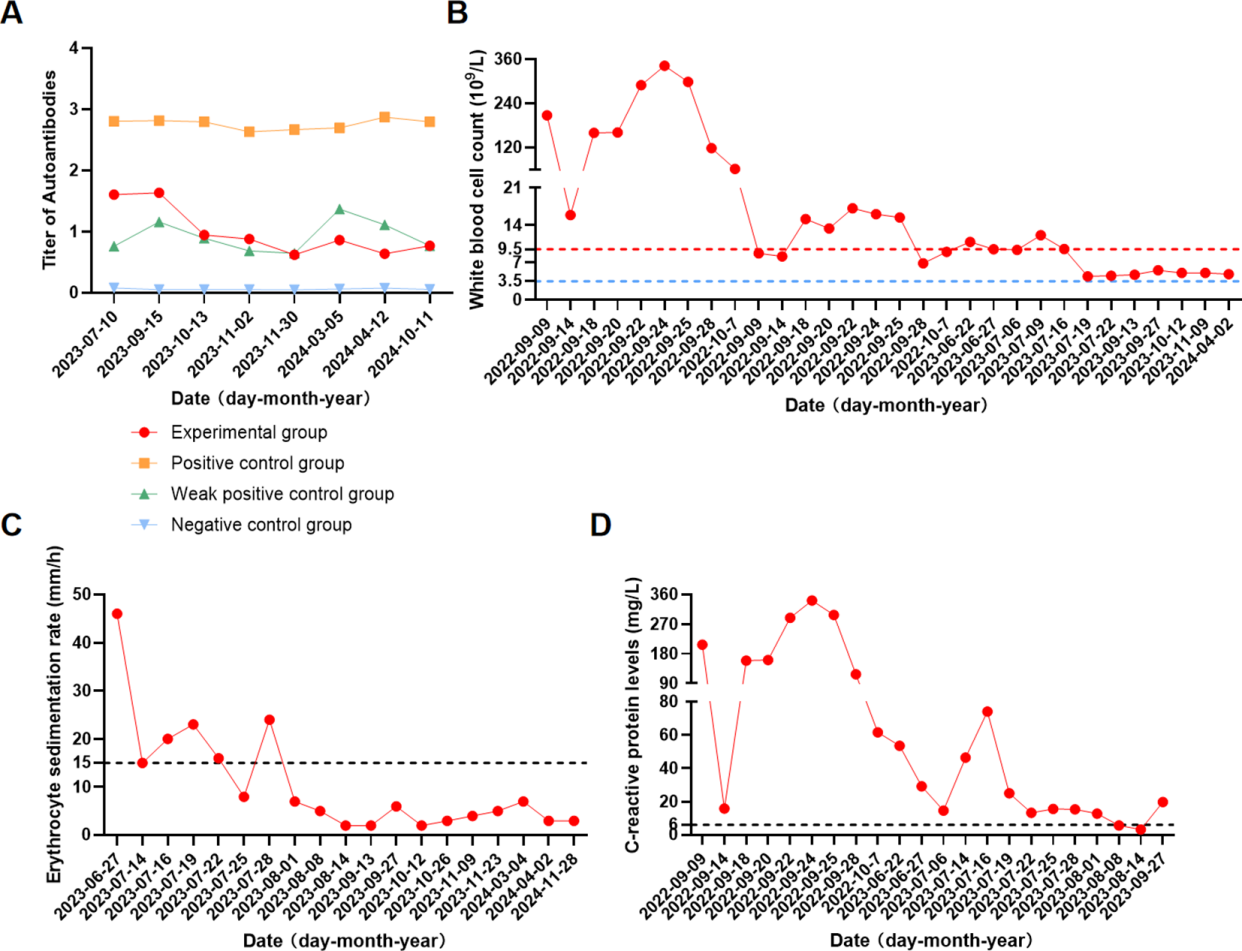
Statistics on the detection of indicators related to anti-gamma interferon autoantibody immunodeficiency syndrome. **(A)** Datas of the patient’s serum AIGA IgG levels (ELISA). The experimental group represents the patient’s serum. Positive, weak positive, and negative controls provided with the ELISA kit indicate high, low, and absent antibody levels, respectively, and serve as assay validation references. OD values were measured according to the manufacturer’s instructions; **(B)** Datas of the patient’s white blood cell (WBC) count levels; **(C)** Datas of the patient’s erythrocyte sedimentation rate (ESR) levels; **(D)** Datas of the patient’s C-reactive protein (CRP) levels. The dashed line represents the normal range.

In September 2023, the patient was referred to the Department of Rheumatology and Immunology for further evaluation, and serial monitoring of serum AIGA levels was initiated. Testing revealed a markedly elevated IgG titer of AIGA (**Figure 4A**). Considering the patient’s history of recurrent infections caused by intracellular opportunistic pathogens and the exclusion of other primary or secondary immunodeficiency disorders, these findings provided definitive etiologic evidence for AOID.

On September 24, 2023, during outpatient follow-up, the patient’s AIGA titer remained elevated, prompting hospital readmission for initiation of long-term immune-targeted therapy with rituximab. The patient reported no history of recent vaccination prior to treatment. Following the first infusion, by September 28, the patient’s peripheral B-cell count had decreased to <20 cells/μL (**Supplementary Table S5**). Concurrently, voriconazole was prescribed for antifungal therapy, while rifampicin and entecavir were administered as prophylaxis against tuberculosis and viral infections, respectively. The patient’s infectious symptoms gradually improved.

After a second rituximab infusion on October 13, 2023, the antibody titer decreased to I = 0.944, and further declined to I = 0.623 by November 30, 2023. However, a mild rebound was observed (I = 0.862) on March 5, 2024 (**Figure 4A**). Consequently, rituximab therapy was continued to deplete B cells and plasma cells, thereby suppressing autoantibody production, enhancing immune recovery, and reducing susceptibility to opportunistic infections. The patient’s WBC, ESR, and CRP levels remained persistently elevated prior to immunotherapy (**Figures 4B–D**), correlating closely with AIGA titers.

By May 16, 2024, the titer remained low positive (I = 0.639), with a slight increase noted on October 11, 2024 (I = 0.769). The patient continued prophylactic antiviral therapy with entecavir and underwent regular clinical and serological monitoring. Longitudinal changes in serum antibody titers are summarized in **Figure 4A**. The patient reported good adherence to treatment and active engagement with the treatment plan, with overall favorable clinical outcomes. However, sleep disturbances became more frequent, and he sought evaluation in the psychiatry department.

## 3 Discussion and conclusions

AIGA is a rare disease with atypical clinical manifestations that are often masked by other clinical infections, resulting in a high rate of misdiagnosis, poor prognosis, and high mortality. Studies have shown that HLA-DRB1*15:01, DRB1*16:02, DQB1*05:01, and DQB1*05:02 alleles are significantly enriched in AIGA-positive patients, supporting a genetic predisposition to disease (9). AIGA is characterized by disseminated and recurrent infections, resembling those seen in immunocompromised HIV patients. A multicenter retrospective study reported that 46.7% of AIGA-positive patients experienced recurrent bacterial infections (6). Common pathogens include *nontuberculous mycobacteria* (e.g., *Mycobacterium abscessus* and *Mycobacterium avium* complex), *Talaromyces marneffei*, and varicella-zoster virus. In contrast, *Candida* and *Pneumocystis carinii* are rarely reported (10, 11). The disease most commonly involves the lymph nodes (up to 80%) and may also affect the bone, lungs, skin, and bloodstream. Current studies have shown a strong association between AIGA and *T. marneffei* infection, which can occur in more than 60% of patients (12, 13). In this case, pathogen identification was based on culture, mNGS, and MALDI-TOF MS. Additional tests were performed to exclude opportunistic infections. These included cryptococcal antigen testing, galactomannan (GM) assay (1, 3),-β-D-glucan testing, interferon-γ release assay (IGRA), and fungal culture.

This report suggests that AIGA–associated infection susceptibility are heterogeneous across individuals, and certain clinical presentations may not adequately reflect the underlying immunodeficient state. *Salmonella enteritidis* is a serotype in the genus *Salmonella*, which mainly causes acute gastroenteritis, leading to fever, diarrhea, abdominal pain, and vomiting in patients. At the initial stage, the infection was not readily linked to an underlying autoimmune condition. Therefore, treatment was limited to symptomatic management, including anti-infective therapy and modulation of intestinal flora. Four days after the initial infection was controlled, the patient developed L. pneumophila infection. *L. pneumophila* is an opportunistic pathogen that can cause severe or fatal pneumonia. Hospital water systems are recognized reservoirs for its persistence (14). The patient’s impaired immune status likely predisposed to infection with *L. pneumophila*, resulting in rapidly progressive and severe pneumonia (**Supplementary Table S2**).

However, given the patient’s age, underlying disease and the timely control of the pneumonia, the infection during the first hospitalization did not raise significant clinical concern. The patient had already experienced restricted right shoulder movement upon first discharge, and the possibility of potential pathogen infection was overlooked. It was not until 8 months later that the patient entered the acute phase of the infection and was re-hospitalization with infectious shoulder arthritis caused by a right shoulder infection with *T. marneffei* (**Supplementary Figure S1**). *T. marneffei* is classified as a medium-priority response by the list of fungal priority pathogens to be released by the WHO in 2022 (15). It is a facultative intracellular pathogen that can invade and survive within mononuclear phagocytes, leading to systemic dissemination (16). Effective host defense against *T. marneffei* relies heavily on IFN-γ–mediated activation of macrophages and Th1 immune responses. AOID can neutralize IFN-γ signaling, impair macrophage activation and intracellular pathogen clearance, and thereby predispose patients to disseminated *T. marneffei* infection. Although *T. marneffei* infection is classically associated with patients with advanced HIV infection (17), HIV infection was excluded in this case. This prompted us to turn our attention to anti cytokine autoantibody syndrome. The serum AIGA IgG was detected by ELISA and continuously tested positive (**Figure 4A**), suggesting the possibility of AOID. Previously reported immunological features of AOID include B-cell dysfunction, elevated immunoglobulins (including IgG and IgE), increased eosinophil count, and elevated levels of other autoantibodies (18, 19). In this case, most of the relevant immune parameters were within normal ranges, except for an elevated total IgE level (**Supplementary Tables S1**, **S4**, **S5**). The patient exhibited a less pronounced immune phenotype, making AIGA more insidious than previously reported cases (**Supplementary Tables S2**, **S6**). Conventional inflammatory markers, including WBC count, ESR, and CRP, are frequently elevated in AIGA-associated AOID and may correlate with disease activity and AIGA titers (20, 21). In this case, the patient’s WBC, ESR, and CRP levels remained persistently elevated prior to immunotherapy (**Figures 4B–D**), paralleling AIGA titers. Following prolonged immunotherapy, these parameters gradually stabilized and declined, further supporting previous reports (20, 21).

Notably, AIGA-associated immunodeficiency is characterized by a selective impairment of IFN-γ–mediated immunity, resulting in increased susceptibility to intracellular pathogens while largely preserving responses to common extracellular microbes, which may contribute to delayed clinical recognition. Although a comprehensive longitudinal cytokine profile was not available, the existing data provide insight into the underlying immune state. During acute infection, IFN-γ–mediated (Th1) immunity was impaired, while inflammatory responses were preserved, as reflected by elevated IL-6. Th2 and immunoregulatory cytokines (e.g., IL-4) were not elevated at this stage (**Supplementary Table S7**). This pattern suggests an immune dissociation, characterized by impaired cell-mediated immunity with relatively preserved inflammatory responses, which may obscure the underlying immunodeficiency and delay clinical recognition. These observations indicate that early assessment of cytokine profiles, particularly Th1/Th2–related cytokines, may aid in identifying underlying immune dysfunction. Given the atypical characteristics of AOID, standard antimicrobial therapy may only provide symptomatic relief (treating the symptoms but not the root cause) and could even increase the risk of severe opportunistic infections such as *T. marneffei (*22). Considering that a prolonged treatment period may increase mortality and recurrence risk, for rare cases of infection with unknown etiology, especially in cases with delayed clinical presentation, priority should be given to detecting potential immune deficiency factors. Currently, no standardized clinical protocol for detecting AIGA has been established. Various methods, including ELISA, protein arrays, and liquid-phase assays, have been applied for detection (23–25). Additionally, the QuantiFERON-TB (QFT) assay, originally designed for tuberculosis screening, can also be used to indirectly assess the neutralizing activity of AIGAs. This method indirectly reflects AIGA neutralizing capacity by measuring the degree of inhibition of IFN-γ release in whole-blood supernatants using ELISA. However, in patients with high-titer AIGAs, the mitogen response may be negative, likely because high levels of AIGA bind to IFN-γ and thereby interfere with the assay, leading to spuriously reduced or even negative results. At present, the optimal cutoff value for defining AIGA neutralizing activity by this method remains uncertain (26, 27). In this case, serum AIGA IgG levels were primarily determined using an indirect ELISA, the most commonly used qualitative assay, and the results were considered reliable. However, ELISA-based detection reflects only antigen–antibody binding and does not directly evaluate the neutralizing activity of the autoantibodies (28). As such functional assays are not currently available at our institution, this remains an important limitation of the present study (9).

There is currently no consensus on the effective treatment of AOID, with evidence based primarily on case reports and small-scale exploratory studies (29). It is well established that antimicrobial therapy alone is insufficient for pathogen clearance in AOID patients and cannot prevent recurrent and polymicrobial infections. Therefore, immune modulation is a key component of treatment and should be administered concurrently with pathogen-targeted antimicrobial therapy. Anti-CD20 monoclonal antibody (rituximab) is the most widely reported immunotherapy for AOID (30). Rituximab reduces circulating B cells, thereby lowering AIGA titers, restoring normal IFN-γ signaling, and improving immune function (6, 30). Cyclophosphamide combined with corticosteroid therapy, plasma exchange, and anti-plasma cell therapies have also been used to treat AOID (6). However, a multicenter study reported that exogenous recombinant IFN-γ, immunoglobulin, and plasma exchange therapies were ineffective, highlighting the need for careful assessment and close monitoring during treatment. If therapy fails, a timely switch to alternative treatments is necessary to avoid disease progression (6). It is also essential to monitor for secondary opportunistic infections caused by treatment-related immunosuppression (31). Proteasome inhibitor bortezomib can be used in the treatment of AOID through depletion of both short-lived and long-lived plasma cells. However, given its limited activity against plasma cell precursors, combination therapy with an anti-CD20 monoclonal antibody such as rituximab may provide broader coverage across different stages of plasma cell development (32).

In this case, the patient received long-term immunotherapy with rituximab. Voriconazole was administered to treat *T. marneffei* infection. In addition, rifampin and entecavir were given prophylactically for tuberculosis and hepatitis B virus reactivation, respectively. This strategy reduced antibody titers and controlled infection, consistent with previous reports (33). Following anti-CD20 therapy, white blood cell and neutrophil counts remained within normal ranges, indicating no significant bone marrow suppression or ongoing infection. Lymphocyte counts showed a transient decrease but gradually recovered, reflecting expected B-cell depletion and immune reconstitution. B cells remained persistently low (0–19/μL), confirming effective depletion. Total T cells remained near the lower limit of normal, with a sustained elevated CD4^+^/CD8^+^ ratio, suggesting preserved cellular immunity. These findings indicate that rituximab depletes B cells and reduces autoantibody production without markedly impairing T-cell function, thereby maintaining immune homeostasis. However, the optimal treatment duration remains unclear, and disease relapse after treatment discontinuation remains a major clinical challenge (18).

In conclusion, this case report describes a highly atypical and insidious presentation of AOID with a prolonged course of recurrent infections. It underscores the importance of measuring serum AIGA levels in patients with recurrent disseminated infections caused by intracellular pathogens, despite normal routine immunological assessments (e.g., HIV-negative status). A comprehensive treatment approach combining targeted antimicrobial therapy, immune modulation, and prophylactic antiviral therapy proves effective in controlling infections while reducing the risk of secondary infections. Enhancing clinical awareness of AOID, establishing early screening protocols, and optimizing treatment strategies are critical for facilitating early intervention and improving patient outcomes.

## Data Availability

The original contributions presented in the study are included in the article/**Supplementary Material**. Further inquiries can be directed to the corresponding authors.
