# Supplementary material for: Anti–interferon-γ autoantibody–associated adult-onset immunodeficiency with occult immunological abnormalities and sequential intracellular infections: a case report
Source: Front Immunol. 2026 May 13;17:1840699. doi: 10.3389/fimmu.2026.1840699 (PMC13212536; doi:10.3389/fimmu.2026.1840699)
Supplement: Supplementary file 1 [file Supplementaryfile1.docx]

**Table S1 Biological parameters of the patient (period 1**^a^**)**

| **Primary Outcome** | **Reference** | **Date** | | | | | | | | |
| --- | --- | --- | --- | --- | --- | --- | --- | --- | --- | --- |
|  |  | **2022-**  **09-09** | **2022-**  **09-14** | **2022-**  **09-18** | **2022-**  **09-20** | **2022-**  **09-22** | **2022-**  **09-24** | **2022-**  **09-25** | **2022-**  **09-28** | **2022-**  **10-07** |
| **Hematological and immune cell parameters** | | | | | | | | | | |
| WBC (10^9^/L) | 3.50-9.50 | 8.74 | 8.15 | 15.15 | 13.38 | 17.17 | 16.04 | 15.43 | 6.84 | 8.98 |
| Neutrophil (10^9^/L) | 1.80-6.30 | 5.64 | 5.91 | **12.88** | **10.81** | **13.52** | **13.31** | **12.38** | 4.52 | **6.43** |
| Lymphocytes (10^9^/L) | 1.10-3.20 | 2.22 | 1.78 | 1.58 | 1.63 | 2.18 | 1.54 | 1.79 | 1.42 | 1.59 |
| Eosinophil  (10^9^/L) | 0.02-0.5 | 0.11 | 0.03 | 0.03 | 0.04 | 0.07 | 0.07 | 0.16 | 0.18 | 0.33 |
| Monocyte  (10^9^/L) | 0.1-0.6 | **0.76** | 0.42 | **0.64** | **0.88** | **1.39** | **1.10** | **1.07** | **0.70** | **0.60** |
| HB (g/L) | 130-175 | 134.00 | **112.00** | **112.00** | **114.00** | **118.00** | **115.00** | **117.00** | **107.00** | **94.00** |
| PLT (10^9^/L) | 125-350 | **112.00**^b^ | 199.00 | 312.00 | 340.00 | **368.00** | 328.00 | 319.00 | 264.00 | 309.00 |
| T cell (CD3^+^)/µL | 797.00-2370.00 |  |  |  |  |  | 1086.00 |  |  |  |
| CD4^+^ T cell/µL | 432.00-1341.00 |  |  |  |  |  | 547.00 |  |  |  |
| CD8^+^ T cell/µL | 238.00-1075.00 |  |  |  |  |  | 548.00 |  |  |  |
| CD4^+^/CD8^+^ | 0.69.00-2.72.00 |  |  |  |  |  | 1.00 |  |  |  |
| B cell (CD3-CD19^+^)/µL | 86.00-594.00 |  |  |  |  |  | 193.00 |  |  |  |
| NK cell (CD3-CD56 CD16^+^)/µL | 127.00-987.00 |  |  |  |  |  | 233.00 |  |  |  |
| **Immunological and inflammatory parameters** | | | | | | | | | | |
| G testing (pg/ml) | 0.00-70.00 | ＜37.50 |  |  |  |  |  |  |  |  |
| IgA (mg/dL) | 1.00-4.20 | 3.58 |  |  |  |  |  |  |  |  |
| IgM (mg/dL) | 0.30-2.20 | 1.17 |  |  |  |  |  |  |  |  |
| IgG (mg/dL) | 8.60-17.40 | 15.85 |  |  |  |  |  |  |  |  |
| T. IgE (IU/mL) | 0.00-105.29 |  |  |  |  |  | **263.85** |  |  |  |
| CRP (mg/L) | 0.00-6.00 | **207.10** | **15.90** | **159.30** | **160.60** | **289.20** | **341.80** | **297.90** | **117.80** | **61.60** |
| PCT (ng/mL) | 0.00-0.50 | **1.72** | 0.12 |  | 0.09 | 0.15 |  | 0.16 | 0.07 | 0.07 |
| **Biochemical parameters** | | | | | | | | | | |
| T. BIL (umol/L) | 0.00-20.00 | 11.00 | 3.00 | 10.00 | 10.00 | 10.00 | 11.00 | 12.00 | 11.00 | 11.00 |
| D. BIL (umol/L) | 3.00-15.00 | 7.00 | 9.00 | 3.00 | 3.00 | 3.00 | 4.00 | 4.00 | 4.00 | 3.00 |
| ALT (U/L) | 9.00-50.00 | 39.00 | 24.00 | 11.00 | 11.00 | **7.00** | **8.00** | **7.00** | **7.00** | **6.00** |
| AST (U/L) | 15.00-40.00 | 18.00 | 21.00 | **12.00** | **12.00** | **12.00** | **13.00** | **12.00** | **14.00** | **12.00** |
| GGT (U/L) | 10.00-60.00 | 45.00 | 53.00 | 32.00 | 24.00 | 23.00 | 20.00 | 21.00 | 19.00 | 22.00 |
| LDH (U/L) | 0.00-247.00 | 232.00 |  |  |  |  | **296.00** | 236.00 | 203.00 |  |
| AKP (U/L) | 45.00-125.00 | 80.00 | 51.00 | 47.00 | **43.00** | 48.00 | 66.00 | 67.00 | 55.00 | 48.00 |
| ALB (g/L) | 40.00-55.00 | **32.40** | **25.40** | **27.90** | **30.30** | **29.20** | **28.60** | **28.40** | **27.60** | **30.30** |
| Glu (g/L) | 20.00-40.00 | 37.50 | 27.00 | 26.50 | 30.30 | 29.40 | 28.40 | 30.10 | 30.30 | 28.30 |
| Urea(mmol/L) | 2.80-7.20 | **12.40** | **2.20** | **2.60** | **2.20** | 4.20 | 5.20 | 5.50 | 3.80 | **2.20** |
| UA (µmol/L) | 208.00-428.00 | **637.00** | **510.00** | **434.00** | 415.00 |  | 274.00 | 294.00 | 228.00 | 244.00 |
| Cr (µmol/L) | 44.00-97.00 | 79.00 | 50.00 | 57.00 | 63.00 |  | 49.00 | 51.00 | 54.00 | 53.00 |
| BUN/Cr Ratio | 15.00-24.00 | **38.90** | **10.90** | **11.30** | **8.70** |  | **26.30** | **26.70** | 17.40 | **10.30** |

**Notes:** ^a^ During hospitalization for *Salmonella enteritidis* infection and *Legionella pneumonia* infection. ^b^ The bolded numbers indicate that they are outside the normal range.

**Abbreviations:** WBC, White Blood Cell; HB, Hemoglobin; PLT, Platelet; G testing, (1,3)-β-D-glucan Testing; IgA, Immunoglobulin A; IgM, Immunoglobulin M; IgG, Immunoglobulin G; T. IgE, Total Immunoglobulin E; CRP, C-Reactive Protein; PCT, Procalcitonin; T. BIL, Total Bilirubin; D. BIL, Direct Bilirubin; ALT, Alanine Aminotransferase; AST, Aspartate Aminotransferase; GGT, Gamma-Glutamyl Transferase; LDH, Lactate Dehydrogenase; AKP, Alkaline Phosphatase; ALB, Albumin; Glu, Glucose; UA, Uric Acid; Cr, Creatinine; BUN/Cr Ratio, Blood Urea Nitrogen to Creatinine Ratio.

**Table S2 Patient's immune status and infection test results (period 1**^a^**)**

| **Primary Outcome** | **Reference** | **Date** | | | | | | |
| --- | --- | --- | --- | --- | --- | --- | --- | --- |
|  |  | **2022-09-09** | **2022-09-12** | **2022-09-20** | **2022-09-23** | **2022-09- 24** | **2022-10-02** | **2022-10-05** |
| *Rubella* virus IgG (IU/mL) | 0.00-9.00 | **11.80**^b^ |  |  |  |  |  |  |
| *Rubella* virus IgM (IU/mL) | 0.00-20.00 | 1.63 |  |  |  |  |  |  |
| *Cytomegalovirus* IgG (IU/mL) | 0.00-12.00 | **127.00** |  |  |  |  |  |  |
| *Cytomegalovirus* IgM (IU/mL) | 0.00-18.00 | 4.09 |  |  |  |  |  |  |
| *Aspergillus* Antigen (IU/mL) | 0.00-0.50 | 0.11 |  |  | 0.10 |  |  |  |
| *Aspergillus* IgG Antibody (AU/mL) | 0.00-80.00 | **378.40** |  |  | **260.53** |  |  |  |
| *Aspergillus Antigen* (Bronchoalveolar lavage fluid) | 0.00-0.80 |  |  |  |  | **1.14** |  |  |
| *Mycoplasma pneumoniae* IgG (AU/mL) | ＜24.00 |  |  |  | 3.10 |  |  |  |
| *Mycoplasma pneumoniae* IgM (AU/mL) | ＜0.90 |  |  |  | 0.14 |  |  |  |
| *Chlamydia pneumoniae* IgG (AU/mL) | ＜20.00 |  |  |  | **28.5** |  |  |  |
| *Chlamydia pneumoniae* IgM (AU/mL) | ＜0.90 |  |  |  | 0.13 |  |  |  |
| *Legionella pneumophila* DNA | - |  |  |  |  | **+** |  |  |
| Fecal culture |  |  | *Salmonella enteritidis* | *Salmonella enteritidis* |  |  |  | None^c^ |
| Sputum culture |  |  |  |  |  |  | *Burkholderia mallei* |  |
| Bronchoalveolar lavage fluid culture |  |  |  |  |  | *Legionella pneumophila* |  |  |
| Blood culture |  |  |  | *Salmonella enteritidis* |  |  |  |  |

^a^ During hospitalization for *Salmonella enteritidis* infection and *Legionella pneumonia* infection.

^b^ The bolded numbers indicate that they are outside the normal range.

^c^ None, No growth of *Shigella* or *Salmonella*

**Table S3 Metagenomic next-generation sequencing (mNGS)**

| **Genus** | | | | **Species** | | |
| --- | --- | --- | --- | --- | --- | --- |
| **Serial number** | **Name** | **Sequence** | **Relative abundance (%)** | **Name** | **Sequence** | **Relative abundance (%)** |
| 1 | *Legionella* | 352 | 2.05 | *Legionella pnenumophila* | 347 | 2.03 |
| 2 | *Rhadinovirus* | 22 | 0.13 | *gammaherpesvirus 8 (Kaposi's sarcoma-associated herpesvirus)* | 22 | 0.13 |
| 3 | *Mycobacteria* | Not detected | | | | |
| 4 | *Mycoplasma/Chlamydia/Rickettsia* | Not detected | | | | |
| 5 | *Fungi* | Not detected | | | | |
| 6 | *RNA viruses* | Not detected | | | | |
| 7 | *Parasites* | Not detected | | | | |
| 8 | *Probable normal flora* | 9499 | 55.30 | *Neisseria mucosa* | 3182 | 18.53 |
|  |  | 2881 | 16.78 | *Lautropia mirabilis* | 2881 | 16.78 |
|  |  | 1308 | 7.62 | *Enterococcus faecalis* | 1293 | 7.53 |
|  |  | 9499 | 55.30 | *Neisseria subflava* | 866 | 5.05 |
|  |  | 507 | 2.96 | *Staphylococcus epidermidis* | 395 | 2.30 |
|  |  | 208 | 1.22 | *Rothia mucilaginosa* | 197 | 1.15 |
|  |  | 67 | 0.40 | *Lacticaseibacillus paracasei* | 62 | 0.37 |
|  |  | 47 | 0.28 | *Mycoplasma orale* | 42 | 0.25 |
|  |  | 21 | 0.13 | *Escherichia coli* | 21 | 0.13 |
|  |  | 13 | 0.08 | *Fusobacterium nucleatum* | 12 | 0.08 |
|  |  | 9 | 0.06 | *Human gammaherpesvirus 4 (Epstein–Barr virus)* | 9 | 0.06 |
|  |  | 21 | 0.13 | *Haemophilus influenzae* | 7 | 0.05 |
|  |  | 3 | 0.02 | *Stenotrophomonas maltophilia* | 2 | 0.02 |
|  |  | 1 | <0.01 | *Human betaherpesvirus 6B* | 1 | <0.01 |
| Q index^a^ | | | | Q index^b^ | | |
| 1 | 13690.36，More than 56.74% of similar specimens | | | 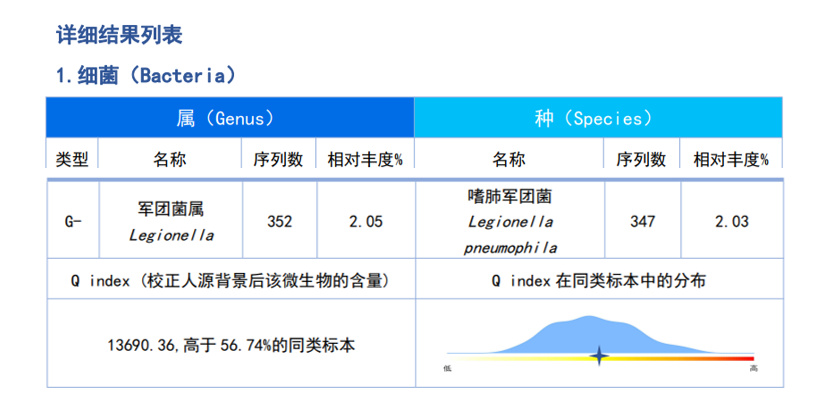 | | |
| 2 | 9712.61，More than 83.65% of similar specimens | | | 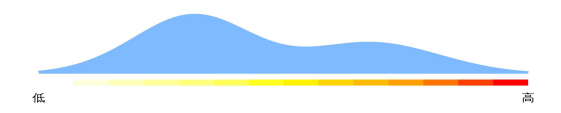 | | |

^a^ The content of the microorganism after correcting the human source background.

^b^ Distribution among similar specimens.

**Table S4 Biological parameters of the patient (period 2**^a^**)**

| **Primary Outcome** | **Reference** | **Date** | | | | | | |
| --- | --- | --- | --- | --- | --- | --- | --- | --- |
|  |  | **2023-**  **06-22** | **2023-**  **06-27** | **2023-**  **07-06** | **2023-**  **07-09** | **2023-**  **07-16** | **2023-**  **07-19** | **2023-**  **07-22** |
| **Hematological and immune cell parameters** | | | | | | | | |
| WBC (10^9^/L) | 3.50-9.50 | **10.88**^b^ | 9.47 | 9.36 | **12.12** | **9.51** | 4.40 | 4.52 |
| Neutrophil (10^9^/L) | 1.80-6.30 | **7.73** | **7.01** | **7.00** | **8.19** | **6.94** | 2.66 | 2.40 |
| Lymphocytes (10^9^/L) | 1.10-3.20 | 1.93 | 1.45 | 1.26 | 2.92 | 1.38 | **1.01** | 1.50 |
| Eosinophil (10^9^/L) | 0.02-0.5 | **0.63** | 0.47 | **0.71** | 0.29 | **0.51** | 0.24 | 0.28 |
| Monocyte (10^9^/L) | 0.1-0.6 | 0.56 | 0.51 | 0.37 | **0.69** | **0.66** | 0.47 | 0.32 |
| HB (g/L) | 130.00-175.00 | **117.00** | **112.00** | **125.00** | **126.00** | **91.00** | **87.00** | **86.00** |
| PLT (10^9^/L) | 125.00-350.00 | 243.00 | 224.00 | 246.00 | 261.00 | 147.00 | 159.00 | 178.00 |
| T cell (CD3^+^)/µL | 797.00-2370.00 |  | 974.00 |  |  |  |  |  |
| CD4^+^ T cell/µL | 432.00-1341.00 |  | 673.00 |  |  |  |  |  |
| CD8^+^ T cell/µL | 238.00-1075.00 |  | 307.00 |  |  |  |  |  |
| CD4^+^/CD8^+^ | 0.69-2.72 |  | 2.19 |  |  |  |  |  |
| B cell (CD3-CD19^+^)/µL | 86.00-594.00 |  | 218.00 |  |  |  |  |  |
| **Immunological and inflammatory parameters** | | | | | | | | |
| G testing (pg/ml) | 0.00-70.00 |  | ＜37.5 | 51.40 |  |  |  |  |
| IgA (mg/dL) | 1.00-4.20 |  | 2.14 |  |  |  |  |  |
| IgM (mg/dL) | 0.30-2.20 |  | 1.08 |  |  |  |  |  |
| IgG (mg/dL) | 8.60-17.40 |  | 13.63 |  |  |  |  |  |
| T. IgE (IU/mL) | 0.00-105.29 |  |  | **144.51** |  |  |  |  |
| CRP (mg/L) | 0.00-6.00 | **53.50** | **29.20** | **14.60** | 4.60 | **74.10** | **25.00** | **13.30** |
| **Biochemical parameters** | | | | | | | | |
| T. BIL (umol/L) | 0.00-20.00 |  | 7.00 | 9.00 |  | 5.00 | 7.00 | ＜5 |
| D. BIL (umol/L) | 3.00-15.00 |  | ＜2 | ＜2 |  | ＜2 | ＜2 | ＜2 |
| ALT (U/L) | 9.00-50.00 |  | 12.00 | 13.00 | 12.00 | **8.00** | **6.00** | 20.00 |
| AST (U/L) | 15.00-40.00 |  | **13.00** | **12.00** | **11.00** | **9.00** | **12.00** | 20.00 |
| GGT (U/L) | 10.00-60.00 |  | 34.00 | 36.00 |  | 32.00 | 49.00 | **74.00** |
| LDH (U/L) | 0.00-247.00 |  | 138.00 | 144.00 |  |  |  |  |
| AKP (U/L) | 45.00-125.00 |  | 64.00 | 75.00 |  | 59.00 | 62.00 | 67.00 |
| ALB (g/L) | 40.00-55.00 |  | 41.50 | 40.70 |  | 30.50 | 31.70 | 33.90 |
| Glu (g/L) | 20.00-40.00 |  | 30.80 | 33.70 |  | 22.80 | 26.30 | 24.50 |
| Urea(mmol/L) | 2.80-7.20 |  | 5.70 | 4.20 | 6.70 | **17.50** | **12.50** | **8.30** |
| UA (µmol/L) | 208.00-428.00 |  | **169.00** | 282.00 |  | **488.00** | **440.00** | 376.00 |
| Cr (µmol/L) | 44.00-97.00 |  | 67.00 | 65.00 |  | **270.00** | **156.00** | **125.00** |
| BUN/Cr Ratio | 15.00-24.00 |  | 21.10 | 16.00 |  | 16.10 | 19.90 | 16.50 |
| NK cell (CD3-CD56 CD16^+^)/µL | 127.00-987.00 |  | 229.00 |  |  |  |  |  |

**Notes:** ^a^ During hospitalization for *Talaromyces marneffei* infection. ^b^ The bolded numbers indicate that they are outside the normal range.

**Abbreviations:** WBC, White Blood Cell; HB, Hemoglobin; PLT, Platelet; G testing, (1,3)-β-D-glucan Testing; IgA, Immunoglobulin A; IgM, Immunoglobulin M; IgG, Immunoglobulin G; T. IgE, Total Immunoglobulin E; CRP, C-Reactive Protein; T. BIL, Total Bilirubin; D. BIL, Direct Bilirubin; ALT, Alanine Aminotransferase; AST, Aspartate Aminotransferase; GGT, Gamma-Glutamyl Transferase; LDH, Lactate Dehydrogenase; AKP, Alkaline Phosphatase; ALB, Albumin; Glu, Glucose; UA, Uric Acid; Cr, Creatinine; BUN/Cr Ratio, Blood Urea Nitrogen to Creatinine Ratio.

**Table S5 Biological parameters of the (period 3**^a^**)**

| **Primary Outcome** | **Reference** | **Date** | | | | | | | |
| --- | --- | --- | --- | --- | --- | --- | --- | --- | --- |
|  |  | **2023-**  **09-13** | **2023-**  **09-27** | **2023-**  **10-12** | **2023-**  **10-26** | **2023-**  **11-09** | **2023-**  **11-23** | **2024-**  **03-04** | **2024-**  **04-02** |
| **Hematological and immune cell parameters** | | | | | | | | | |
| WBC (10^9^/L) | 3.50-9.50 | 4.71 | 5.55 | 5.05 |  | 5.07 |  |  | 4.82 |
| Neutrophil (10^9^/L) | 1.80-6.30 | 2.45 | 2.45 | 3.50 |  | 3.48 |  |  | 3.21 |
| Lymphocytes (10^9^/L) | 1.10-3.20 | 1.57 | 2.23 | **0.98** | 1.36 | **1.05** | **2.01** | 2.25 | **1.06** |
| Eosinophil (10^9^/L) | 0.02-0.5 | 0.36 | 0.43 | 0.23 |  | 0.17 |  |  | 0.21 |
| Monocyte (10^9^/L) | 0.1-0.6 | 0.31 | 0.40 | 0.30 |  | 0.34 |  |  | 0.32 |
| HB (g/L) | 130.00-175.00 | **105.00**^b^ | **109.00** | **112.00** |  | **113.00** |  |  | **119.00** |
| PLT (10^9^/L) | 125.00-350.00 | 136.00 | 148.00 | 167.00 |  | 159.00 |  |  | 137.00 |
| T cell (CD3^+^)/µL | 797.00-2370.00 | 980.00 | 1200.00 | **774.00** | 938.00 | 805.00 | 1150.00 | 1067.00 | 834.00 |
| CD4^+^ T cell/µL | 432.00-1341.00 | 686.00 | 823.00 | 562.00 | 706.00 | 591.00 | 802.00 | 740.00 | 612.00 |
| CD8^+^ T cell/µL | 238.00-1075.00 | 311.00 | 377.00 | **201.00** | **235.00** | **226.00** | 350.00 | 340.00 | **218.00** |
| CD4^+^/CD8^+^ | 0.69-2.72 | 2.21 | 2.18 | **2.80** | **3.00** | 2.62 | 2.29 | 2.18 | **2.80** |
| B cell (CD3-CD19^+^)/µL | 86.00-594.00 | 242.00 | 252.00 | **0.00** | **3.00** | **0.00** | **0.00** | **2.00** | **19.00** |
| NK cell (CD3-CD56CD16^+^)/µL | 127.00-987.00 | 350.00 | 758.00 | 190.00 | 394.00 | 240.00 | 808.00 | **1163.00** | 197.00 |
| **Immunological and inflammatory parameters** | | | | | | | | | |
| IgA (g/L) | 1.00-4.20 | 2.22 | 1.93 |  |  |  |  |  |  |
| IgM (g/L) | 0.30-2.20 | 1.55 | 1.48 |  |  |  |  |  |  |
| IgG (g/L) | 8.60-17.40 | 12.60 | 11.19 |  |  |  |  |  |  |
| IgG4 (g/L) | 0.03-2.01 | 1.56 |  |  |  |  |  |  |  |
| C3 | 0.70-1.40 | 1.36 | 1.37 |  |  |  |  |  |  |
| C4 | 0.10-0.40 | 0.20 | 0.28 |  |  |  |  |  |  |
| CRP (mg/L) | 0.00-6.00 | ＜1.0 | **19.70** |  |  |  |  |  |  |
| **Biochemical parameters** | | | | | | | | | |
| T. BIL (umol/L) | 0.00-20.00 | 5.00 | 6.00 |  |  |  |  |  |  |
| D. BIL (umol/L) | 3.00-15.00 | ＜2 | ＜2 |  |  |  |  |  |  |
| ALT (U/L) | 9.00-50.00 | **66.00** | **67.00** |  |  |  |  |  |  |
| AST (U/L) | 15.00-40.00 | 26.00 | 32.00 |  |  |  |  |  |  |
| GGT (U/L) | 10.00-60.00 | 60.00 | **62.00** |  |  |  |  |  |  |
| LDH (U/L) | 0.00-247.00 | 129.00 | 138.00 |  |  |  |  |  |  |
| AKP (U/L) | 45.00-125.00 | 53.00 | 55.00 |  |  |  |  |  |  |
| ALB (g/L) | 40.00-55.00 | 43.10 | 42.30 |  |  |  |  |  |  |
| Glo (g/L) | 20.00-40.00 | 24.50 | 27.40 |  |  |  |  |  |  |
| Glu (mmol/L) | 3.90-6.10 | 4.80 | **6.80** |  |  |  |  |  |  |
| Urea (mmol/L) | 2.80-7.20 | **8.20** | 6.90 |  |  |  |  |  |  |
| UA (µmol/L) | 208.00-428.00 | 426.00 | 360.00 |  |  |  |  |  |  |
| Cr (µmol/L) | 44.00-97.00 | 86.00 | 86.00 |  |  |  |  |  |  |
| BUN/Cr Ratio | 15.00-24.00 | 23.60 | 19.90 |  |  |  |  |  |  |

**Notes:** ^a^ During immunotherapy. ^b^ The bolded numbers indicate that they are outside the normal range.

**Abbreviations:** WBC, White Blood Cell; HB, Hemoglobin; PLT, Platelet; IgA, Immunoglobulin A; IgM, Immunoglobulin M; IgG, Immunoglobulin G; IgG4, Immunoglobulin G4; C3, Complement Component 3; C4, Complement Component 4; CRP, C-Reactive Protein; T. BIL, Total Bilirubin; D. BIL, Direct Bilirubin; ALT, Alanine Aminotransferase; AST, Aspartate Aminotransferase; GGT, Gamma-Glutamyl Transferase; LDH, Lactate Dehydrogenase; AKP, Alkaline Phosphatase; ALB, Albumin; Glo, Globulin; Glu, Glucose; UA, Uric Acid; Cr, Creatinine; BUN/Cr Ratio, Blood Urea Nitrogen to Creatinine Ra

**Table S6 Patient's immune status and infection test results (period 3**^a^**)**

|  | **Reference** | **2023-06-27** | **2023-06-29** | **2023-07-02** | **2023-07-06** | **2023-07-12** |
| --- | --- | --- | --- | --- | --- | --- |
| T. IgE (IU/mL) | 0.00-105.29 |  |  |  | **144.51**^b^ |  |
| Aspergillus-specificsIgE (M3) | 0.00-0.35 |  |  |  | ＜0.10 |  |
| Aspergillus Antigen (IU/mL) | 0.00-0.50 | 0.05 |  |  | 0.07 |  |
| Aspergillus IgG Antibody (AU/mL) | 0.00-80.00 | 40.39 |  |  |  |  |
| Drainage Fluid Culture |  |  | *Talaromyces marneffei* |  |  |  |
| Pus Culture |  |  |  | *Talaromyces marneffei* |  |  |
| Wound Exudate Culture |  |  |  |  |  | *Talaromyces marneffei* |
| Joint Fluid Culture |  |  |  |  |  | *Talaromyces marneffei* |

^a^ During hospitalization for *Talaromyces marneffei* infection.

^b^ The bolded numbers indicate that they are outside the normal range.

**Table S7 Cytokine Changes in the Patient**

| **Primary Outcome** | **Reference** | **Date** | | | |
| --- | --- | --- | --- | --- | --- |
|  |  | **2022-09-23** | **2023-06-27** | **2023-07-06** | **2024-04-02** |
| IL-2 (pg/mL) | ＜6.13 | 0.10 | 0.10 | 0.05 | ＜2.25 |
| IL-4 (pg/mL) | ＜4.72 | 0.10 | 0.95 | 1.95 | **5.21** |
| IL-6 (pg/mL) | ＜6.28 | **124.29** | **7.92** | 4.33 | 3.33 |
| IL-10 (pg/mL) | ＜6.09 | 0.10 | **8.64** | 5.91 | ＜2.5 |
| TNF-α (pg/mL) | ＜5.12 | 2.35 | 0.51 | 1.01 | ＜2.5 |
| IFN-γ (pg/mL) | ＜6.77 | 0.10 | 1.02 | 1.27 | 5.31 |
| IL-8 (pg/mL) | ＜15.56 |  |  |  | **19.75** |

**Abbreviations:** IL, interleukin; TNF-α, tumor necrosis factor alpha; IFN-γ, interferon gamma.

*
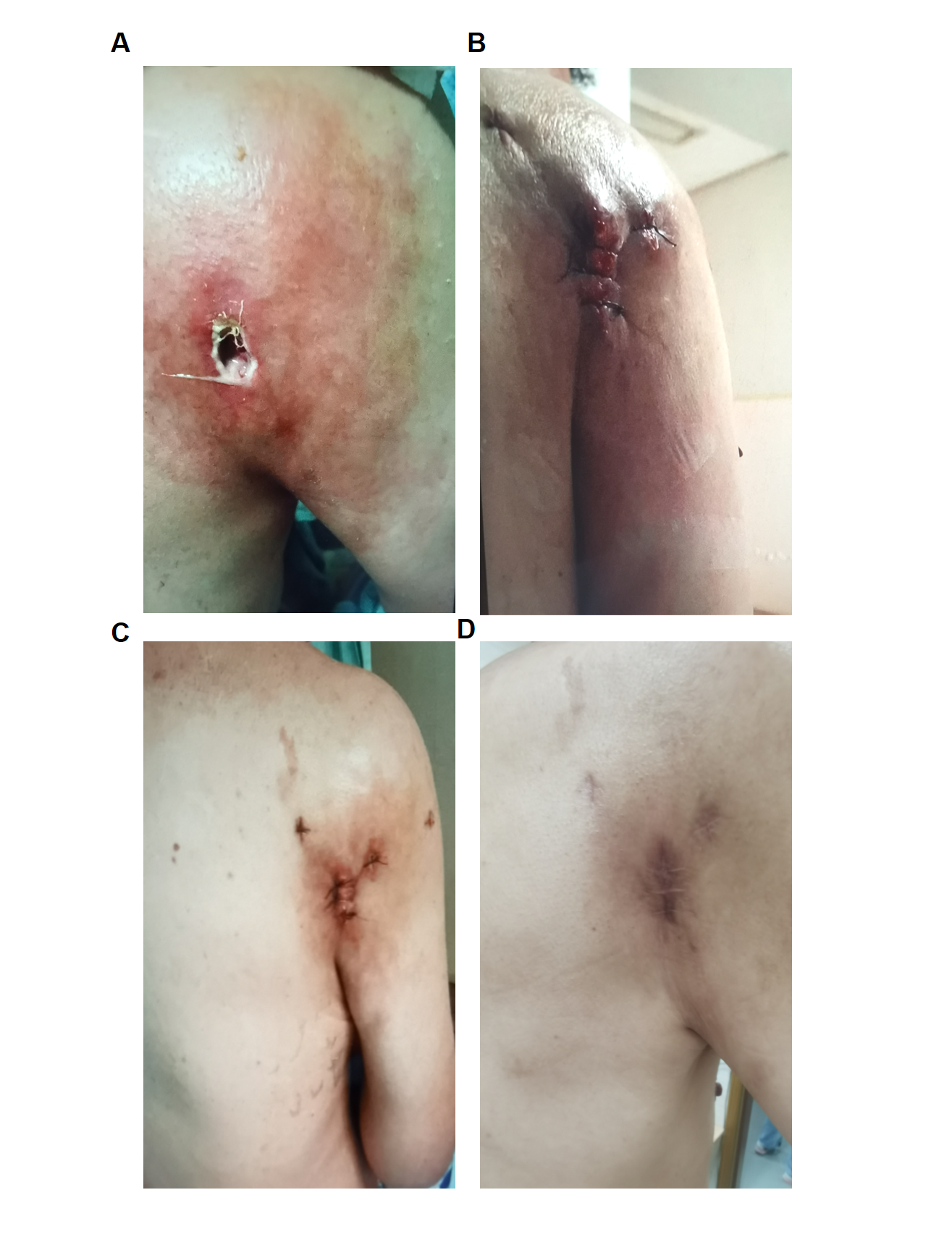
*

**Figure S1 Wound progression of right shoulder infection caused by *Talaromyces marneffei*.**

(A) Abscess formation in the right shoulder due to infection, with poor response to empirical antibacterial therapy. (B) Arthroscopic debridement of the right shoulder abscess was performed to control the infection. (C) The infection was effectively controlled following antifungal therapy. (D) Complete wound healing with satisfactory recovery.
